# Probiotics, postbiotics, and synbiotics in immune system modulation: A narrative review

**DOI:** 10.1016/j.biotno.2026.06.001

**Published:** 2026-06-24

**Authors:** Mina Owrang

**Affiliations:** Department of Medical Laboratory Sciences, Faculty of Medical Sciences, Sari.C., Islamic Azad University, Sari, Iran

**Keywords:** Microbiota, Immune system, Probiotic, Postbiotic, Synbioti

## Abstract

Interactions between the intestinal microbiota and the immune system play a crucial role in human health and the pathogenesis of various diseases. Since many beneficial effects of probiotics are mediated through their bioactive metabolites, increasing attention has been directed toward postbiotics and synbiotics as potential alternatives or complementary microbiota-based interventions. This review summarizes current evidence regarding the interactions and immunomodulatory effects of probiotics, postbiotics, and synbiotics on the human immune system. Although the immunomodulatory effects of probiotics have been extensively investigated, evidence regarding synbiotics and postbiotics remains comparatively limited and continues to evolve. A deeper understanding of the interactions between microbiota-derived bioactives and the immune system may contribute to the development of novel therapeutic strategies. This study was conducted as a narrative review based on a qualitative synthesis of the currently available literature.

## 1 Introduction

Modulation of the immune system has become a major focus of contemporary biomedical research. The primary function of the immune system is to protect the host against pathogenic invasion through mechanisms of pathogen recognition described by the danger theory: “stranger” (pathogen-associated molecular patterns-PAMPs) and “danger” (damage-associated molecular patterns-DAMPs).^1^, ^2^, ^3^ The immune system exerts its protective functions through two major defense mechanisms: innate and adaptive immunity.^1^ The innate immune system has physical barriers such as skin, mucous membranes, and endothelia throughout the body that prevent the entry of invasive microorganisms into the host body with potential sites of infection.^4^ In addition, these physical barriers are colonized by symbiotic commensal microorganisms located on epithelial surfaces such as the gastrointestinal tract and skin.^5^ Collectively referred to as the microbiota, these microorganisms play essential roles in maintaining host physiological homeostasis.^6^ Therefore, maintaining a balanced symbiotic relationship between the host immune system and the intestinal microbiota is essential for human health.^7^ The intestinal microbiota of the human body called intestinal flora has a vital role in human health and regulation of the host immune system.^8^ Considerable interindividual variability exists in the composition of intestinal microbial communities. Because intestinal microbiota composition is closely associated with immune homeostasis and overall health, interest in microbiota-targeted interventions such as probiotics, prebiotics, and synbiotics has markedly increased.^9^ According to the definition of the International Scientific Association for Probiotic and Prebiotic (ISAPP), probiotics are living microorganisms with health benefits for the host body if they have been taken in sufficient amounts.^10^^,^^11^ On the other hand, synbiotics are living microorganisms mixed with the substrate (s) selectively used by host microorganisms, and have beneficial effects on host health.^12^ Several non-viable microbial preparations, including paraprobiotics, ghost probiotics, and metabiotics, that do not conform to the ISAPP definition of probiotics are classified under the broader category of postbiotics.^13^ Previous studies have suggested that prebiotics, synbiotics, and postbiotics may exert beneficial effects on host health and immune system regulation.^14^ The immunomodulatory effects of these bioactive compounds are mediated through maintenance of intestinal mucosal barrier integrity and stimulation of both innate and adaptive immune responses.^15^ Elucidating the immunological mechanisms underlying the activity of these bioactive compounds remains an important and rapidly evolving area of research. The aim of this review is to provide a comprehensive and integrative narrative review of probiotics, postbiotics, and synbiotics, with particular emphasis on their comparative roles and underlying mechanisms in modulating the human immune system. Despite the rapidly expanding literature on microbiota-related interventions, several limitations remain in the current review landscape. Most currently available reviews have examined probiotics, prebiotics, or synbiotics separately, or have primarily emphasized clinical outcomes without providing a comprehensive mechanistic comparison.^16^ In particular, the role of postbiotics, defined as non-viable microbial products with biological activity, has only recently gained attention and remains insufficiently explored in the context of immune modulation. Therefore, this review aims to address this gap by providing an integrated and comparative analysis of probiotics, postbiotics, and synbiotics, with a specific focus on their interactions with the human immune system. This study further emphasizes underlying immunological mechanisms, including cytokine regulation, immune cell modulation, and microbial metabolite activity. By synthesizing recent findings and highlighting emerging concepts such as paraprobiotics and metabiotics, this review offers a more comprehensive and up-to-date perspective on microbiota-based strategies for immune regulation.

## 2 Narrative review approach

A structured literature search was conducted to identify relevant studies addressing the immunomodulatory effects of probiotics, postbiotics, and synbiotics. Relevant literature published up to January 1, 2025, was systematically searched, and the final search was completed on January 15, 2025. Studies evaluating the effects of probiotics, synbiotics, and postbiotics, including in vitro studies and human clinical *(in vivo)* studies, were considered. Relevant studies were identified through searches of Scopus, PubMed/MEDLINE, Google Scholar, and ClinicalTrials.gov databases. The following keywords and combinations thereof were used as search terms: "probiotic”, “synbiotics”, “postbiotics ”,” ghost probiotics”, “metabiotics” were combined with “human immune system”, “anti-inflammatory”, “inflammatory”, OR “immune regulatory” to be used as search terms. The inclusion criteria were studies involving the effect of probiotics, synbiotics, postbiotics on immune cells, cytokines, or other immune factors. Studies were screened based on relevance to the scope of the review, with particular emphasis on investigations addressing immune modulation, cytokine regulation, and microbiota-related mechanisms. Following title and abstract screening, the most relevant and scientifically informative studies were selected for qualitative synthesis. Although a structured literature search strategy was employed, the selection and synthesis of studies were not performed according to a predefined systematic review protocol; therefore, the findings should be interpreted as a qualitative narrative synthesis rather than a quantitative systematic analysis. In this review, the term ‘*in vivo*’ refers exclusively to human clinical studies, and animal studies were not considered.

## 3 Interactions of intestinal microbiota and immune system

The human body maintains a symbiotic relationship with a vast community of microorganisms, whose large number live in the intestine.^17^ The human gastrointestinal tract contains more than 40 trillion microbial cells,f with a small proportion of fungus and Protista.^18^ There is a distinct diversity in bacterial strains among individuals. The host genotype determines unique intestinal microbiota via initial colonization through vertical transmission at birth and dietary habits.^19^^,^^20^ For the first time, the Human Microbiome Project (HMP) that examines the interaction of the microbiota with genetics, gender, age, nutrition, drugs, and environmental factors suggested the connection between human health and microbiota.^21^ Increasing evidence supporting the fundamental role of the microbiota in the induction and function of the human immune system opened a new avenue in the field of immunology.^22^ A complex network of innate and adaptive components makes the immune system with a high capacity to adapt and respond to highly diverse challenges. The development of this complex network of the immune system, especially adaptive immunity, has coincided with the acquisition of a complex microbiota. In other words, a large fraction of the immune system has evolved to maintain synbiotic relationships with these highly diverse microbiotas.^23^^,^^24^ Having an immune system with complex and precise functions and its reliance on the microbiota can be costly at the same time. Dysregulation of host–microbiota interactions has been associated with autoimmune and inflammatory disorders, against microbiota-derived or environmental antigens, and the pathologies such as allergies.^25^^,^^26^

**Fig. 1:**
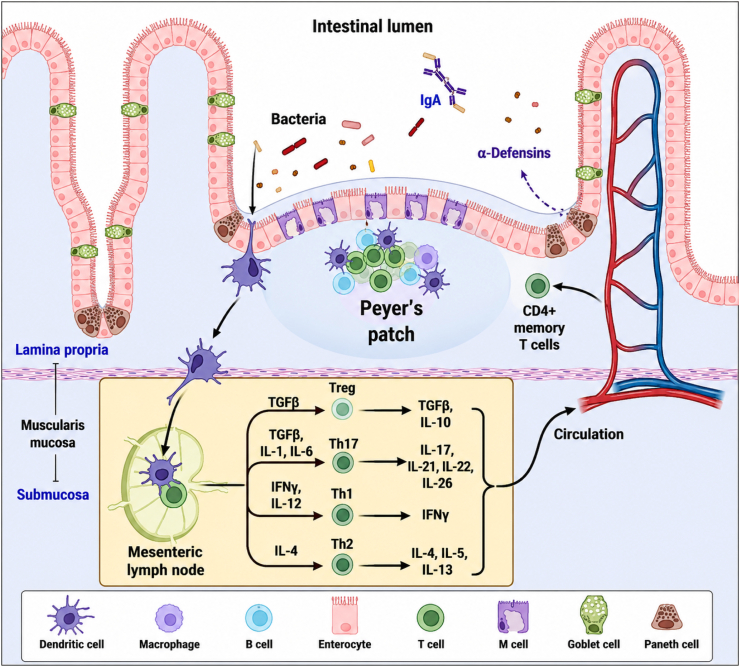
Schematic illustration of the interactions between intestinal microbiota and host immune system components involved in mucosal immune regulation.

The fetal gastrointestinal tract has traditionally been considered sterile, and the early interactions of microbiota community with the mucosal and systemic immune system is remained unknown.^22^ Breastfeeding with milk rich in maternal antibodies, live microorganisms, and bioactive metabolites facilitates passive transfer of immunity from mother to infan.^27^ Previous studies demonstrated that maternal bioactive compounds are necessary for the modulation of infant health and the development of their immune system with a unique microbiota.^28^ The live microbes in the breast milk are composed of *Staphylococcus*, *Veillonella, Gemella*, *Enterococcus*, *Streptococcus*, *Bifidobacterium*, *Lactobacillus, Propioni*, *Actinomyces*, *Clostridia, Pseudomonas, Sphingomonas*, *Corynebacterium, Serratia*, *Enterobacter*, *Escherichia*, and *Ralstonia*, *Bradyrhizobium* and *Prevotella*.^29^^,^^30^ The development of the immune response, the intestinal-associated lymphoid tissue (GALT), the innate immune system, and the adaptive immune system coincides with the development of intestinal microbiota.^31^ Interactions between intestinal microbiota and immune cells involve highly complex regulatory mechanisms (Fig. 1). It seems that intestinal epithelial cells (IECs) secrete mucins and AMPs in response to the microbiota to limit microbial interaction with these cells.^32^ The microbe-associated molecular patterns (MAMPs) in the IECs under homeostatic and eubiotic conditions can stimulate the secretion of cytokines such as TSLP, IL-33, IL-25, and TGFβ. Then, these cytokines promote the development of tolerogenic dendritic cells and macrophages.^27^ The TGFβ- and retinoic acid (RA)- the dependent process of dendritic cells can induce the development of induced Treg (iTreg) cells. The anti-inflammatory balance of the intestine is maintained via multiple mechanisms including the secretion of IL-10 by macrophages and TGFβ and IL-10 by iTreg cells inhibiting or dampening potential effector responses. In addition, the epithelial-derived BAFF and APRIL, Treg cell-derived TGFβ promote development of IgA + plasma cells that result in abundant supply of sIgA in the lumen and restricted microbial interaction with the epithelium.^27^^,^^31^^,^^32^

## 4 Interactions of probiotics and the immune system

The term "probiotic" is derived from the Greek word meaning "for life." According to ISAPP, probiotics are defined as live microorganisms that, when administered in adequate amounts, confer a health benefit on the host. The three most extensively studied probiotic genera in humans are Lactobacillus, Bifidobacterium, and Saccharomyces.^33^ Although numerous studies have reported immunomodulatory effects of probiotics, the observed outcomes remain partially inconsistent across experimental and clinical settings. Such discrepancies may result from variations in probiotic strains, host physiological conditions, dosage regimens, and study design.^34^^,^^35^ Therefore, a critical evaluation of these variables is necessary to better understand the variability in immune responses.The presence of probiotics in the intestine can promote the balance of T-cells and Treg cells against pathogens via multiple mechanisms that prevent ectopic colonization of invasive bacteria (such as *Klebsiella*). Inducing phagocytosis activity of dendritic cells and macrophages by probiotics plays a vital role in antibacterial immune response.^36^^,^^37^ On the other hand, the Treg cells under the modulator effect of probiotics secrete molecules such as IL-10 and TGF-β which exert suppressive action on immune cells.^38^ However, the extent and direction of these immune responses appear to be strain-specific, and in some contexts, probiotics may also induce pro-inflammatory signaling, highlighting the importance of considering strain-dependent functional differences.

Probiotics affect the expression of multiple intestinal genes such as stimulatory molecules (CD80, CD86, and CD40) in dendritic cells to enhance the intestinal mucosal barrier.^39^^,^^40^ Notably, microbial metabolites may exert both beneficial and context-dependent effects on immune regulation. For instance, while indole derivatives are generally associated with anti-inflammatory properties, their influence on pathways such as NF-κB signaling may vary depending on concentration and host cellular context.^41^ This duality may partially explain the heterogeneity observed across experimental studies.

Some previous studies showed that probiotics can regulate host immune response through the production of a series of metabolites by digesting different foods.^42^^,^^43^ The amino acid metabolites, particularly tryptophan (Trp), produced as metabolites of probiotics, are closely related to the immune system.^44^^,^^45^ Indole, one of the major bacterial tryptophan metabolites.^41^ Trp metabolites can induce TNF-α expression via activation of the NF-κB pathway and reduce the expression of IL-8, a pro-inflammatory chemokine. In addition, Indol can reduce the adhesive capacity of pathogenic *E. coli* to HCT- 8 cells.^44^ Probiotic-mediated synthesis of vitamins, particularly B-group vitamins can promote intestinal immune reactions such as lymphocytes migration to the periphery, activation of MHC class II molecules on immune cell, activation of mucosal-associated invariant T cells (MAITs), and secretion of IL-17 and IFN γ.^46^, ^47^, ^48^

The intestinal probiotics can produce Short-Chain Fatty Acids (SCFA) through the fermentation of fibers. SCFAs exert both extracellular and intracellular immunoregulatory functions.^49^ In extracellular signaling pathways, SCFAs act as ligands for G-protein-coupled receptors.^50^ During the intracellular pathway, SCFAs can regulate the transcription of genes encoding inflammatory mediators, including IL-6 and IL-12, through inhibition of histone deacetylases (HDACs).^51^ Despite the well-established immunoregulatory roles of SCFAs, inconsistencies remain in translating these findings into clinical outcomes. Such discrepancies may arise from variations in microbiota composition, dietary background, and individual host responses, underscoring the complexity of microbiota–immune system interactions.^52^^,^^53^ Overall, while substantial evidence supports the role of probiotics in immune modulation, the variability in study outcomes highlights the need for a more nuanced and context-specific understanding. Future research should aim to standardize experimental conditions and focus on strain-specific effects, host–microbiome interactions, and well-designed clinical trials to clarify the mechanisms underlying these diverse immunological responses.

## 5 Interactions of postbiotics and the immune system

Increasing scientific evidence suggests that microbial vitality is not required to achieve the health benefits of probiotic supplements. Consequently, postbiotics derived from probiotic microorganisms may confer health benefits similar to those associated with live probiotics.^54^ Postbiotics are defined as preparations of inanimate microorganisms and/or their components that confer health benefits to the host.^13^ According to this definition, postbiotics can be broadly categorized into microbial cell components (e.g., cell wall fragments and peptidoglycans), metabolites (e.g., short-chain fatty acids), and secreted bioactive compounds, including extracellular polysaccharides (EPS) and functional proteins. Some previous studies reported that the immunomodulatory activity of postbiotics produced via inactivation of probiotics was higher than probiotics.^55^ Therefore, the postbiotics derived from probiotics may be promising alternative supplements with potentially fewer safety concerns than live probiotic administration. The most common postbiotic components include short-chain fatty acids (SCFAs), microbial cell fractions, extracellular polysaccharides (EPS), functional proteins, cell lysates, and other bioactive metabolites such as flavonoids and phenolic-derived compounds.^56^, ^57^, ^58^ It seems that the molecular mechanism underlying the effects of postbiotics on the host immune system is mediated through an interaction between the host and microbial products.^59^ Most studies investigating the immunological mechanisms of postbiotics have been limited to in *vitro* experimental models.; Therefore, the precise immunological mechanisms of the prebiotics leading to the health benefits in humans have not been fully elucidated.^55^

Among these components, extracellular polysaccharides (EPS) have been particularly studied for their role in modulating immune responses through interactions with host immune receptors.^59^ Postbiotics can exert an immunomodulatory effect and protect the body against pathogenic bacterial invasion by the protection of the intestinal barrier, factor proteins, promoting aggregation, S-layer proteins, and bacteriocins.^60^ It appears that some postbiotics such as lipoteichoic acid and peptidoglycan can increase Th1-associated cytokine levels and decrease Th2-related cytokines.^61^ Also, these postbiotics protect intestinal cells from pathogen infection by improving membrane integrity via modulating cytokine gene expression.^15^

Previous studies demonstrated that postbiotics can protect the gastric mucosa through modulation of anti-inflammatory response such as cytokine IL-8 production.^62^ However, the current understanding of postbiotic mechanisms remains limited, as most available evidence is derived from in vitro studies. The lack of well-controlled human clinical trials makes it difficult to draw definitive conclusions regarding their immunological efficacy. This gap represents an important area for future investigation. Accumulating evidence suggests that postbiotics may exert beneficial immunomodulatory effects, therefore, these bioactive molecules may be safe alternatives for immunocompromised individuals such as premature neonates, elderly, and transplanted patients. However, the assurance of their benefits can only be determined through clinical studies and the determination of precise functional mechanisms in the future.

## 6 Interactions of synbiotics and the immune system

The combination of a probiotic microorganism with a prebiotic compound that selectively promotes its growth constitutes what is termed a symbiotic.^63^ The use of synbiotics can have multiple and different influences on the host immune system. However, the precise mechanisms underlying the immunological activity of synbiotics and the differences in their function with probiotics and postbiotics is at the beginning of the research path.^64^

Synbiotics may exert antimicrobial effects through multiple mechanisms, including modulation of gut microbiota composition and stimulation of antimicrobial peptide production Several studies have suggested that these mechanisms are highly context-dependent.^65^^,^^66^ Evidence from clinical and experimental studies suggests that synbiotic supplementation—typically consisting of specific probiotic strains such as *Lactobacillus* or *Bifidobacterium* combined with prebiotic substrates such as inulin or fructooligosaccharides, may improve gut microbial balance and modulate immune and inflammatory parameters, including high-sensitivity C-reactive protein, FOXP3^+^ regulatory T cells, and IL-17 levels. These effects have been reported in both healthy individuals and patient populations, including those with inflammatory or metabolic disorders. In several studies, synbiotics have been administered through functional food matrices (e.g., fermented dairy products or synbiotic-enriched formulations), which may further influence their bioavailability and immunological impact. However, the magnitude and direction of these effects vary depending on factors such as formulation composition, dosage, duration of intervention, and host baseline characteristics.^67^^,^^68^ The corresponding clinical trials (NCT01226212, NCT03494036) are provided for reference.

## 7 The risk and disadvantages of probiotics, post biotic, and synbiotic for immune health

In recent years, increasing attention has been directed toward the potential adverse effects associated with microbiota-based interventions. Probiotic consumption may occasionally result in mild gastrointestinal symptoms, such as bloating, gas, or diarrhea. These adverse effects are generally mild, transient, and self-limiting, resolving spontaneously without intervention. However, rare instances exist where individuals with compromised immune systems or underlying health conditions may experience more severe complications. Overall, probiotics are generally considered to have a favorable safety profile. Nevertheless, caution should be exercised, especially concerning sensitive populations.^69^ Although probiotics are live microorganisms, they have the potential to cause systemic infections, harmful metabolic activities, excessive immune response in certain individuals, and gene transfer. Nevertheless, reported adverse events associated with probiotic administration in humans remain relatively rare, with only a limited number of reported cases.^70^ Probiotics have been investigated for their potential impact on dyslipidemia, although not classified as a conventional side effect. A study demonstrated that dyslipidemic patients who consumed probiotic-enriched vegetable and fruit juice containing *Lactobacillus paracasei* experienced significant reductions in total cholesterol, low-density lipoprotein cholesterol, triglycerides, and triglyceride/high-density lipoprotein cholesterol ratio. These findings indicate a potential advantage for managing dyslipidemia.^71^ In addition, the postbiotic and synbiotics caused the production of deconjugated bile salts that result in the weakening of the immune defense against cholestasis or colorectal cancer.^3^^,^^72^ A study evaluating synbiotic ice cream, which includes prebiotic and probiotic components, revealed no noteworthy variance in ice cream consumption between the placebo and treatment phases. Similarly, there were no significant alterations in reported flatulence levels or frequency of bowel movements when comparing the treatment group to the control group. These findings imply that synbiotic ice cream could serve as a suitable vehicle for delivering probiotic ingredients without causing clinically significant gastrointestinal adverse events.^73^

## 8 Conclusions

In recent years, the understanding of the intestinal microenvironment has emerged as a central focus of contemporary immunological research. Interactions between intestinal microbiota and immune system components contribute substantially to both health maintenance and disease pathogenesis. Consequently, scientific and commercial interest in microbiota-targeted interventions has increased considerably. Nevertheless, the immunoregulatory mechanisms underlying these bioactive compounds remain incompletely understood. Due to their biological heterogeneity and variability in clinical outcomes. Future research should focus on the effect of probiotics, postbiotics, and synbiotics on the components of the immune system *in vivo* and clinical study to suggest possible new immune treatments and clarify their potential adverse effects and long-term safety profiles. Looking forward, progress in this field will strongly depend on coordinated collaboration among industry, academia, and research institutes. From an industrial perspective, future work should prioritize the development of standardized, stable, and scalable formulations of probiotic, postbiotic, and synbiotic products, supported by robust quality-control and manufacturing frameworks. Academic research will continue to play a crucial role in elucidating the mechanistic pathways, such as cytokine regulation, immune-cell modulation, and microbial metabolite signaling, that underpin their immunomodulatory effects. At the translational level, integrated industry–academia partnerships are essential for designing rigorous clinical studies, evaluating safety and efficacy in real-world settings, and ensuring regulatory compliance. By bridging these sectors, future investigations can accelerate the development of microbiota-based interventions with practical value for food science, immunotherapy, and public health.

## Declaration of competing interest

The authors declare that they have no known competing financial interests or personal relationships that could have appeared to influence the work reported in this paper.
